# Heat Stress Affects Facultative Symbiont-Mediated Protection from a Parasitoid Wasp

**DOI:** 10.1371/journal.pone.0167180

**Published:** 2016-11-22

**Authors:** Eleanor R. Heyworth, Julia Ferrari

**Affiliations:** Department of Biology, University of York, York, United Kingdom; Pennsylvania State University, UNITED STATES

## Abstract

Many insects carry facultative bacterial symbionts, which provide benefits including resistance to natural enemies and abiotic stresses. Little is known about how these beneficial phenotypes are affected when biotic or abiotic threats occur simultaneously. The pea aphid (*Acyrthosiphon pisum*) can host several well-characterized symbiont species. The symbiont known as X-type can protect against both parasitoid wasps and heat stress. Here, we used three pea aphid genotypes that were naturally infected with X-type and the symbiont *Spiroplasma* sp. We compared aphids coinfected with these two symbionts with those cured from X-type and infected with only *Spiroplasma* to investigate the ability of X-type to confer benefits to the host when two threats are experienced simultaneously. Our aim is to explore how robust symbiont protection may be outside a benign laboratory environment. Aphids were subjected to heat shock either before or after attack by parasitoid wasps. Under a benign temperature regime, the aphids carrying X-type tended to be better protected from the parasitoid than those cured. When the aphids experienced a heat shock before being parasitized aphids carrying X-type were more susceptible than those cured. Regardless of infection with the symbiont, the aphids benefitted from being heat shocked after parasitization. The results demonstrate how resistance to parasitoid wasps can be strongly environment-dependent and that a beneficial phenotype conferred by a symbiont under controlled conditions in the laboratory does not necessarily equate to a consistently useful effect in natural populations.

## Introduction

Insects face many threats to their survival, ranging from the challenges of extreme abiotic conditions such as high temperatures to a wide range of natural enemies. These challenges are rarely encountered in isolation in the natural environment, and the interactions between these different stresses can be the determining factors in insect survival. The ecology and efficiency of natural enemies can be affected by temperature and precipitation [1–4] as well as by the presence of competing species, indirectly affecting the victim [5]. As the surrounding environment affects natural enemy efficacy, it also changes the selection pressures on insect populations.

Many insects depend on their facultative symbionts to increase their survival against common threats, and an increasing number of mutualistic microbes have been shown to provide protection [6–8]. Many such symbionts are vertically transmitted, thus host and microbe fitness are closely linked, and increased insect survival benefits both partners. Facultative symbionts across many taxa can guard against natural enemy attack [9–11] and against abiotic stresses [12,13]. High predation pressure or extreme temperatures may select for insects harboring certain symbionts, and in turn, symbionts can potentially affect the frequencies at which natural enemies are encountered [14,15].

Studying the effects of symbionts on just one trait may miss more complex interactions, and here we used the pea aphid (*Acyrthosiphon pisum*) as a model species to investigate how temperature can affect known symbiont-mediated protection against parasitoid wasps. Pea aphids can be infected with at least eight different species of facultative endosymbionts [16,17]. Several of their symbionts have been shown to ameliorate the negative effects of heat on their hosts [18–21] thus increasing insect reproduction and so symbiont spread into the next generation. Others can protect against mortality due to attack by parasitoids [18,22–24]. Typically, each species of aphid endosymbiont is well known for providing one specific benefit to its host; for example *Hamiltonella defensa* increases resistance to parasitoid wasps [25], *Regiella insecticola* protects against a fungal pathogen [26] and *Serratia symbiotica* increases reproduction and survival after heat stress [20]. In reality, all of these symbiont species have been shown to provide multiple benefits, with potentially more benefits to be found [21,22,24].

The symbiont known as X-type or PAXS (for pea aphid X-type symbiont) is one of the more recently discovered symbionts [27]. It is unusual in that a single isolate has been shown to provide more than one benefit [18], with some isolates improving both tolerance of heat shock and resistance to natural enemies, namely the parasitoid wasp *Aphidius ervi* and the fungal pathogen *Pandora neoaphidis*. As such, it is an ideal symbiont to study how interactions between these abiotic and biotic dangers affect infected aphids.

Previous studies have found that protection from parasitoids provided by *H*. *defensa* can often (but not universally) fail under moderate heat stress [27–29]. Interestingly, in a correlative study comparing pea aphid genotypes that carried natural double infections of *H*. *defensa* and X-type with genotypes that carried only single infections of *H*. *defensa*, those with the double infection maintained most of their resistance to a parasitoid when exposed to heat stress during their development whereas those with single infections were no longer protected under the same conditions [27]. It is therefore possible that the resistance to parasitoids provided by X-type is not affected by temperature extremes, possibly because X-type also confers resistance to heat [18].

We investigated how the protection from the parasitoid *Aphidius ervi* provided by the facultative symbiont X-type is affected by heat stress to illustrate how multiple symbiont-conferred phenotypes interact and to explore the ecological relevance of lab-based symbiont assays under less benign conditions. We employed three pea aphid genotypes that were naturally coinfected with X-type and *Spiroplasma* sp. and selectively removed X-type from these lines, creating a total of six aphid lines. We have previously shown that the lines harboring X-type are more tolerant to heat stress and more resistant to *A*. *ervi* [18].

Facultative symbiont coinfections are common in aphids and X-type is rarely found in single infections [30,31]. The presence of a second symbiont potentially changes the phenotypic effect seen in a single infection, possibly because two symbionts consume more resources [22]. *Spiroplasma* in aphids can confer resistance to a fungal pathogen [32,33] and can affect the host’s fecundity [33–35] particularly in coinfections with *H*. *defensa* [33]. It is therefore possible that the effects we observed in this study are caused by an interaction between *Spiroplasma* and X-type rather than a direct effect of X-type. Regardless of whether this is a direct or indirect effect, the differences within each set of lines will be caused by the presence of X-type.

Based on our previous results [18] and Guay et al.’s [27] observations we hypothesized that (i) X-type protects pea aphids from the parasitoid *Aphidius ervi* under benign conditions and that (ii) this protection is maintained when the aphids experience a heat shock either before or after being parasitized.

## Materials and Methods

### Ethics Statement

The aphids were collected on private land, and we thank the land owners for their permission to sample on their fields. The field work did not involve endangered or protected species.

### Aphids

Pea aphids (*Acyrthosiphon pisum* (Harris)) reproduce asexually under long-day light conditions, allowing genetically identical clonal lines to be maintained in the laboratory. For this study we used the same three pea aphid genotypes (codes 217, 322 and 324) as in our previous characterization of the phenotypic effects of X-type [18]. All three were collected from the UK and naturally infected with X-type and *Spiroplasma*. Genotypes 322 and 324 were collected in 2008 from *Trifolium pratense* and genotype 217 from *Medicago sativa* in 2010, all in Southern England.

The aphids were cured from X-type by feeding them on *Vicia faba* leaves placed in an antibiotic solution of 1% Ampicillin, 0.5% Gentamicin and 0.5% Cefotaxime [36], leading to a total of six aphid lines. These were maintained for at least six months, approximately 12 aphid generations, before the start of the experiment. *Spiroplasma* cannot be removed by this method, so was maintained in all lines. The aphids were also tested for the pea aphid symbionts *H*. *defensa*, *R*. *insecticola*, *S*. *symbiotica*, *Rickettsia* sp. and *Rickettsiella viridis* using symbiont-specific PCR [30,37], and none of these were detected. The aphid lines were retested regularly to confirm infection and ensure that no cross-contamination occurred.

Each pair of uninfected and infected aphid lines of a different genotype might potentially also contain a different strain of the primary symbiont *Buchnera aphidicola* and of *Spiroplasma*, and for simplicity we refer to this combination as ‘aphid background’. We exercise caution when interpreting our results since any observed effect of the presence of X-type may be caused by X-type itself, or by an interaction between the X-type and one or more of the other species involved. Based on the sequences of six household genes, there is little variation between isolates of X-type and no variation has been found for the three isolates used here [38].

Pea aphids of different genotypes generally perform well on *Vicia faba* (L.) [39] and these experiments were all performed using *V*. *faba* (cv. “The Sutton”) leaves or seedlings. Unless otherwise noted, experiments were performed at 20°C and long-day conditions of 16h:8h light:dark with a relative humidity of 40 ± 15% in the controlled temperature room. Aphids were kept in simultaneously refreshed cultures and were raised in small groups prior to use in experiments to reduce maternal effects.

### Susceptibility to a parasitoid under heat stress

To investigate the effects of heat on symbiont-mediated protection against parasitoids, aphids were heat stressed either before or after being exposed to the parasitoid *Aphidius ervi* Haliday. The three heat treatments (heat shock before parasitism, heat shock after parasitism, plus a control that was never heat shocked) allowed us to compare how heat shock affects symbiont mediated resistance to parasitoids at different stages, both before the aphid was attacked and once the parasitoid eggs were laid.

Aphids in all treatments were kept at 20°C, 40 ± 15% relative humidity, and a 16h light: 8h dark regime, except on the day they were heat stressed. To produce the experimental aphids of a standardized age, young adult aphids were placed on *V*. *faba* leaves stuck into 2% agar gel in Petri dishes (Sterilin, 90 mm diameter) overnight. For each replicate, 30 of these offspring were transferred onto *V*. *faba* seedlings. In all three treatments, the aphids were exposed to one female *A*. *ervi* parasitoid for 9 hours at 20°C, starting when they were 72-96h old. On the day of the heat shock treatment, the aphids were exposed to a temperature that steadily rose from 20°C to 37°C over a period of two hours, was stable at 37°C for four hours and then decreased over another two hours back to 20°C. Aphids in the “heat shock before parasitism” treatment experienced this regime when they were 48-72h old (i.e. on the day before being exposed to the parasitoid) and aphids in the “heat shock after parasitism” treatment when they were 96-118h old (i.e. on the day after exposure to the parasitoid). In the control treatment, the aphids were kept at 20°C throughout. For all three treatments, the aphids were transferred to fresh plants on the day after the second heat treatment to minimize the effects that the heat might have had on plant quality.

Successful parasitism involves the parasitoid wasp larva internally consuming the aphid. While the aphid is consumed its cuticle is transformed into a protective casing for the developing wasp, the distinctive brown ‘mummy’. The number of mummies was counted 10 days after exposure to wasps. We also recorded the number of live aphids. Dead, live and mummified aphids were easily identified, but some aphids were not found either at the first transfer to a new plant or at the final count. These aphids were classed as 'disappeared', possibly due to death at a young age where they would have decomposed quickly. We therefore analyzed three outcomes 10 days after exposure to the parasitoid: the number of aphids that had disappeared or were found dead, the number of mummies and the number of aphids that were alive. There were between four and six replicates for each of the six aphid lines in each of the three treatments, stratified across two temporal blocks.

Assays similar to the one described above are routinely used to measure susceptibility to parasitoids (e.g. [25,40,41]). It is possible that the parasitoid exhibited different oviposition behavior in the different treatments, but typically this is observed in choice rather than no-choice assays [25,40,42,43].

### Statistical analysis

The data were analyzed using the R software v. 3.2.1 [44]. We analyzed each outcome employing analysis of deviance, assuming a quasipoisson error distribution. The explanatory variables were heat treatment, aphid background, the presence of X-type and all possible interactions, as well as temporal block. Block was included in the model, but never significant (*P* > 0.1), which we will not report further. The model assumptions were checked with Shapiro-Wilk normality tests. We performed post-hoc tests, using Holm’s correction for multiple testing in the package “phia” in the R software, when an explanatory variable with three levels or an interaction was significant.

## Results

**Fig 1:**
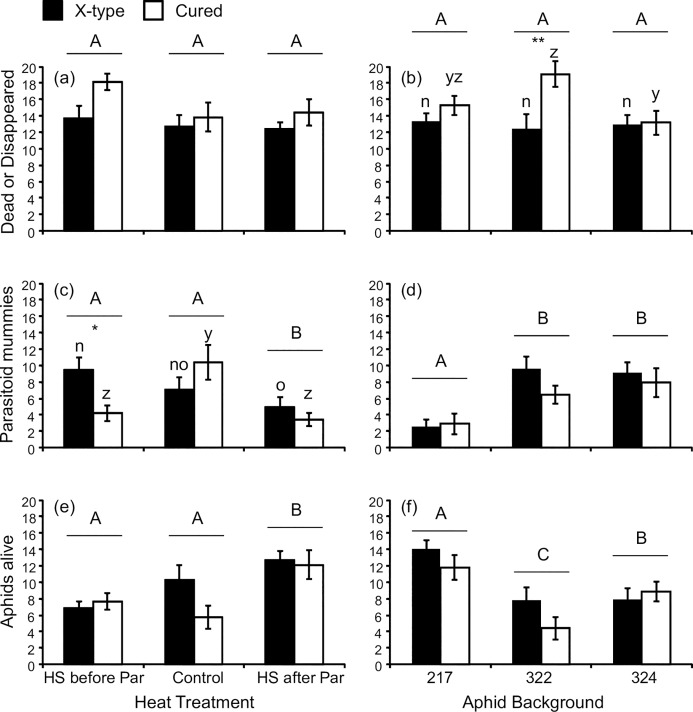
Effects of the Presence of the Facultative Symbiont X-type, Heat Shock and Aphid Background on the Number of Dead or Disappeared Aphids, the Susceptibility to the Parasitoid *Aphidius ervi*, Or Aphid Survival. (a and b) The number of aphids that were dead or had disappeared ten days after parasitoid attack. (c and d) The number of parasitoid mummies ten days after parasitoid attack. (e and f) The number of aphids that were alive ten days after parasitoid attack. All panels show comparisons between aphids that are naturally infected with X-type and *Spiroplasma* (black bars) and those cured of X-type but still infected with *Spiroplasma* (white bars). Means and standard errors are shown. Different capital letters denote significant differences between heat treatments (a, c, e) or between the aphid backgrounds (b, d, f). Different lowercase letters show differences between heat treatments for only the aphids carrying X-type (i.e. between the black bars, post-hoc tests: n and o) or for only the cured aphids (i.e. between the white bars, post-hoc tests: y and z) The asterisks show significant differences between lines infected with X-type and cured from X-type within heat treatments (a, c, e) or within aphid backgrounds (b, d, f) (* *P* < 0.05, ** *P* < 0.01).

We expected some aphids to die initially because of the exposure to heat shock before we could measure the success of the parasitoid. The number of dead or disappeared aphids was relatively high, but did not differ between the control and the two heat shock treatments (Fig 1A, Table 1; *F*_2, 70_ = 2.47, *P* = 0.09). There was no effect of aphid background on the number of dead or disappeared aphids (Fig 1B; *F*_2, 70_ = 2.02, *P* = 0.14). Overall, the presence of X-type reduced the number of dead or disappeared aphids (*F*_1, 70_ = 6.78, *P* = 0.01) which was mainly due to its effect in aphid background 322 (Fig 1B; aphid background × X-type presence: *F*_2, 70_ = 3.38, *P* = 0.04). None of the interactions including heat treatment were significant (Table 1).

**Table 1:** Analysis of Deviance of the Number of Aphids that were Dead or had Disappeared Ten Days after Parasitization.

Explanatory variable	d.f.	Deviance	*F*	*P*
Block	1	4.83	2.70	0.10
Heat Treatment	2	8.83	2.47	0.09
Aphid Background	2	7.24	2.02	0.14
X-type	1	12.11	6.78	0.01
Heat Treatment × Aphid Background	4	11.69	1.63	0.18
Heat Treatment × X-type	2	1.62	0.45	0.64
Aphid Background × X-type	2	12.08	3.38	0.04
Heat Treatment × Aphid Background × X-type	4	9.72	1.36	0.26
Error	70	131.73		

The number of parasitoid mummies was lowest when the aphids experienced heat shock on the day after being parasitized (Fig 1C, Table 2; *F*_2, 70_ = 7.01, *P* = 0.002). It also differed between aphid backgrounds with background 217 being the most resistant as previously observed (Fig 1D; *F*_2, 70_ = 17.34, *P* < 0.001). The presence of X-type had no overall effect on the number of mummies (*F*_1, 70_ = 1.94, *P* = 0.17), but this differed between the heat treatments (Fig 1C; *F*_2, 70_ = 5.62, *P* = 0.005): there was no significant difference between aphids with X-type and those cured when the aphids were heat shocked after parasitoid attack or in the control treatment. However, the aphids carrying X-type were more susceptible than those that did not when heat shocked before parasitoid attack. Compared to the control treatment at 20°C, this difference was due to a decrease of the number of mummies in the aphids that were cured from X-type. There were no significant interactions between aphid background and the other factors (Table 2).

**Table 2:** Analysis of Deviance of the Number of Aphids that had Formed Mummies Ten Days after Parasitization.

Explanatory variable	d.f.	Deviance	*F*	*P*
Block	1	8.80	2.75	0.10
Heat Treatment	2	44.84	7.01	0.002
Aphid Background	2	110.97	17.34	< 0.001
X-type	1	6.20	1.94	0.17
Heat Treatment × Aphid Background	4	25.16	1.97	0.11
Heat Treatment × X-type	2	36.00	5.62	0.005
Aphid Background × X-type	2	5.55	0.87	0.42
Heat Treatment × Aphid Background × X-type	4	13.85	1.08	0.37
Error	70			

When the aphids were heat shocked after parasitoid attack aphid survival was higher than in the other heat treatments (Fig 1E, Table 3; *F*_2, 70_ = 8.41, *P* < 0.001). There was also a difference between aphid backgrounds with aphid background 217 having the highest numbers of survivors and 322 the lowest (Fig 1F; *F*_2, 70_ = 12.25, *P* < 0.001). X-type had no overall effect on aphid survival (*F*_1, 70_ = 1.90, *P* = 0.17) and nor were there any significant interactions between X-type and any of the other explanatory variables (Table 3). There was however a non-significant trend for higher survival of the aphids carrying X-type compared to the cured aphids in the control treatment (heat treatment × X-type: *F*_2, 70_ = 2.51, *P* = 0.09; post-hoc test for this comparison: *P* = 0.053).

**Table 3:** Analysis of Deviance of the Number of Aphids that had Survived Ten Days after Parasitization.

Explanatory variable	d.f.	Deviance	*F*	*P*
Block	1	0.07	0.02	0.88
Heat Treatment	2	46.42	8.41	< 0.001
Aphid Background	2	67.60	12.25	< 0.001
X-type	1	5.23	1.90	0.17
Heat Treatment × Aphid Background	4	7.57	0.69	0.60
Heat Treatment × X-type	2	13.86	2.51	0.09
Aphid Background × X-type	2	11.68	2.11	0.13
Heat Treatment × Aphid Background × X-type	4	5.28	0.48	0.75
Error	70	197.53		

## Discussion

We found that multiple factors can affect an aphid’s susceptibility to parasitism, including the presence of the symbiont X-type, heat stress and aphid background (a composite of aphid genotype, *Buchnera* genotype and *Spiroplasma* genotype). Aphids that were heat shocked after being parasitized were more resistant to the parasitoid than those that experienced the heat shock before parasitoid attack or those in the control treatment. The facultative symbiont X-type tended to confer protection from the parasitoid under the benign temperature regime, which is a benefit of carrying X-type that disappeared in both heat shock treatments.

One caveat of the experiment is that a high number of aphids died or disappeared. This did not differ between the heat treatments, so it is unlikely that it has affected the qualitative patterns but as a precaution we analyzed parasitoid success in two different ways, first as the number of parasitoid mummies (presented in the main manuscript) and second as a proportion of mummies of only the aphids that had not disappeared or died of unknown causes (presented as supplemental material). These two analyses are complementary and show very similar patterns, but differ slightly in the significance of some post-hoc comparisons.

As observed previously [18,45] we found that aphid genotype affects an aphid’s susceptibility to parasitism. Each aphid genotype used here also carries a potentially different strain of *Buchnera* and *Spiroplasma*, so that the observed variation may be due to any of the three species, or indeed an interaction between multiple genotypes (see also [18]).

We previously showed that X-type provided protection from *A*. *ervi* under a benign temperature regime [18], and here we confirm the same trend although this is marginally non-significant (note that this is significant in the supplemental analysis of the proportion of parasitoid mummies; S1 Text, S1 Fig, S1 Table). However, when the aphids were heat stressed after being parasitized, resistance to the parasitoid increased regardless of symbiont infection. At this stage the parasitoid egg is developing inside the aphids, and it is likely that the parasitoid egg is being detrimentally affected by the temperature spike, leading to a decrease in successful development. The fitness of *A*. *ervi* reduces at higher temperatures [46] and the developing egg may have been killed outright, leading to high aphid resistance. The aphids have greater survival in this situation and thus benefit from being exposed to two stresses (parasitoids and heat after parasitoid attack) compared to parasitoid attack on its own. It is however probable that the aphids’ fecundity will be reduced due to the heat shock [21] or the parasitoid [47] later in their life.

When the aphids were heat stressed before being parasitized, aphids cured from X-type produced fewer parasitoid mummies, but aphid survival did not differ between aphids with and without X-type. Instead, the decreased number of mummies in the cured aphids appears to be correlated with a non-significant increase in dead or disappeared aphids (note that while the heat treatment × X-type interaction is not significant, the post-hoc test for this specific comparison is significant, *P* = 0.03). We hypothesize that these cured aphids tended to die or disappear more often than those with X-type, because they did not benefit from X-type’s protection from heat shock [18]. It is possible that the surviving cured aphids are also weaker and therefore not able to support the development of a parasitoid larva or that the parasitoid female’s oviposition behavior might be altered in response to the condition of the aphids. Cayetano and Vorburger [29] observed a similar effect in the black bean aphid *Aphis fabae*: when the aphids were exposed to extreme heat of 39°C before being attacked by the parasitoid *Lysiphlebus fabarum* fewer parasitoid mummies formed at the higher temperature than when they were kept at 20°C. In our system, the presence of X-type appears to prevent this detrimental effect of heat; in aphids carrying X-type the number of parasitoids did not differ between the control and the “heat shock before parasitism” treatment. When the heat shock occurred before parasitoid attack, the presence of X-type thus benefited the parasitoid rather than the aphid.

Our results contrast with Guay et al.’s [27] work where protection from *A*. *ervi* after heat stress was higher in two aphid genotypes that carried a natural coinfection *of H*. *defensa* and X-type than in genotypes naturally infected with only *H*. *defensa*. Our results suggest that the pattern observed by Guay et al. [27] was not a direct effect of X-type, but may have been an effect of the particular aphid [45] or symbiont genotypes [48] involved. We can rule out this explanation in our experiments because our pairs of lines were genetically identical and differed only in the presence of X-type. Alternatively, Guay et al.’s [27] observation may have been an attribute of the coinfection; it is possible that X-type enhanced the protection from parasitoids conferred by *H*. *defensa* rather than being able to protect directly. Similarly, it is possible that in our aphid line X-type is interacting with *Spiroplasma*. *Spiroplasma* can provide resistance to parasitoids in *Drosophila* [10] and it is thus possible that X-type increases resistance to parasitoids that is conferred by *Spiroplasma* in the control treatment. However, the more straightforward explanation is that X-type is able to confer resistance to parasitoids itself.

These results have implications for the understanding of how complex interactions may occur in field populations. There, parasitoid wasps are a common natural enemy [49,50], but aphids and other insects must also face a range of simultaneous threats to their survival. Temperature and precipitation can affect the strength of pathogen and predation pressures, as well as affect the insect itself [46,51]. While the host’s benefit from carrying the X-type symbiont when only one threat is encountered [18], we show here that these benefits can be rendered obsolete when two threats occur simultaneously, and even benefit the parasitoid under certain conditions. This therefore illustrates further that costs and benefits of harboring facultative symbionts can be strongly dependent on the environment and that complex and potentially quite specific interactions can affect the value of a symbiont to an insect host. As facultative symbionts may drive rapid adaptation in host populations due to their protective effects [52], understanding more about how robust symbiont-mediated protection is under different temperature conditions is also vital to understanding how insect populations may be affected by changes in climate in future.

## Supporting Information

S1 Fig **Effects of the Presence of the Facultative Symbiont X-type and (a) Heat Shock or (b) Aphid Background on the Proportion of Aphids that had Formed Mummies out of the Number of Aphids Where one Partner (Aphid or Parasitoid) was Alive Ten Days after Parasitization.** All panels show comparisons between aphids that are naturally infected with X-type and *Spiroplasma* (black bars) and those cured of X-type but still infected with *Spiroplasma* (white bars). Means and standard errors are shown. Different capital letters denote significant differences between heat treatments (a) or between the aphid backgrounds (b). Different lowercase letters show differences between heat treatments for only the aphids carrying X-type (i.e. between the black bars, post-hoc tests: n and o) or for only the cured aphids (i.e. between the white bars, post-hoc tests: y and z) The asterisk shows a significant difference between lines infected with X-type and cured from X-type within a given heat treatments (*P* < 0.05).
(PDF)

S1 Table Analysis of Deviance of the Number of Aphids that had Formed Mummies out of the Number of Aphids Where one Partner (Aphid or Parasitoid) was Alive Ten Days after Parasitization. (PDF)

S1 Text Supplementary Analysis of the Proportion of Parasitoid Mummies. (PDF)
