# Supplementary figures and images for: Heat Stress Affects Facultative Symbiont-Mediated Protection from a Parasitoid Wasp
Source: PLoS One. 2016 Nov 22;11(11):e0167180. doi: 10.1371/journal.pone.0167180 (PMC5119854; doi:10.1371/journal.pone.0167180)

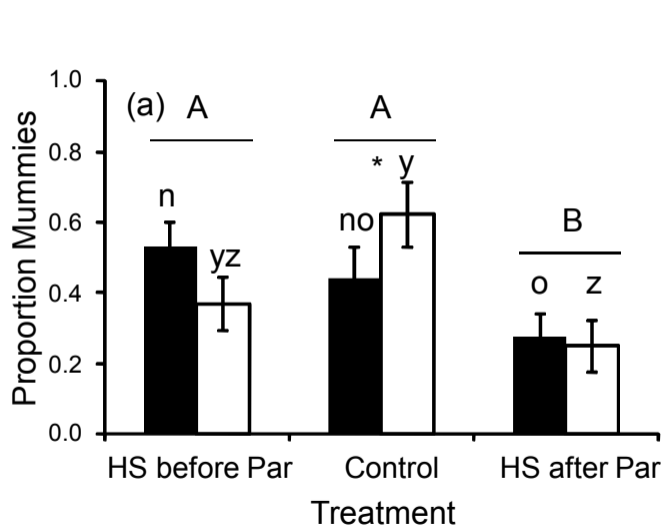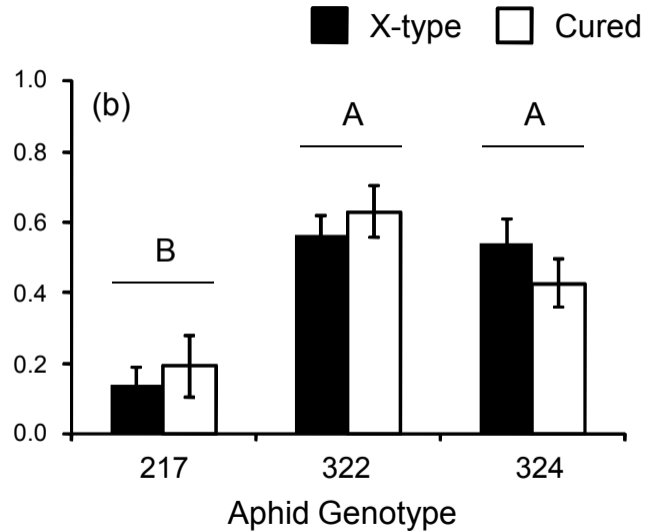

Supplement: S1 Fig — Effects of the Presence of the Facultative Symbiont X-type and (a) Heat Shock or (b) Aphid Background on the Proportion of Aphids that had Formed Mummies out of the Number of Aphids Where one Partner (Aphid or Parasitoid) was Alive Ten Days after Parasitization. All panels show comparisons between aphids that are naturally infected with X-type and Spiroplasma (black bars) and those cured of X-type but still infected with Spiroplasma (white bars). Means and standard errors are shown. Different capital letters denote significant differences between heat treatments (a) or between the aphid backgrounds (b). Different lowercase letters show differences between heat treatments for only the aphids carrying X-type (i.e. between the black bars, post-hoc tests: n and o) or for only the cured aphids (i.e. between the white bars, post-hoc tests: y and z) The asterisk shows a significant difference between lines infected with X-type and cured from X-type within a given heat treatments (P < 0.05). (PDF) [file pone.0167180.s001.pdf]
