# Supplementary material for: Heat Stress Affects Facultative Symbiont-Mediated Protection from a Parasitoid Wasp
Source: PLoS One. 2016 Nov 22;11(11):e0167180. doi: 10.1371/journal.pone.0167180 (PMC5119854; doi:10.1371/journal.pone.0167180)
Supplement: S1 Table — (PDF) [file pone.0167180.s002.pdf]

Supporting Information

Eleanor R. Heyworth, Julia Ferrari

Heat stress affects facultative symbiont-mediated protection from a parasitoid wasp

S1 Table. Analysis of Deviance of the Number of Aphids that had Formed Mummies out of the Number of Aphids Where one Partner (Aphid or Parasitoid) was Alive Ten Days after Parasitization.

Explanatory variable	d.f.	Deviance	*F*	*P*
Block	1	4.46	1.08	0.30
Heat Treatment	2	82.17	9.93	< 0.001
Aphid Background	2	178.67	21.59	< 0.001
X-type	1	0.04	0.01	0.93
Heat Treatment × Aphid Background	4	16.09	0.97	0.43
Heat Treatment × X-type	2	41.25	4.98	0.01
Aphid Background × X-type	2	10.66	1.29	0.28
Heat Treatment × Aphid Background × X-type	4	12.61	0.76	0.55
Error	70	318.89		
