# Supplementary material for: Heat Stress Affects Facultative Symbiont-Mediated Protection from a Parasitoid Wasp
Source: PLoS One. 2016 Nov 22;11(11):e0167180. doi: 10.1371/journal.pone.0167180 (PMC5119854; doi:10.1371/journal.pone.0167180)
Supplement: S1 Text — (PDF) [file pone.0167180.s003.pdf]

Supporting Information

Eleanor R. Heyworth, Julia Ferrari

Heat stress affects facultative symbiont-mediated protection from a parasitoid wasp

S1 Text. Supplementary Analysis of the Proportion of Parasitoid Mummies

The high number of dead or disappeared aphids that we observed in our assays might have obscured some meaningful patterns, and we therefore analyzed parasitoid success in two different ways. The first is presented in the main manuscript (total number of parasitoid mummies). We also analyzed the proportion of mummies formed out of the total numbers of aphids where either the aphid or parasitoid was alive (number of mummies/(live aphids + mummies)), thus excluding the disappeared aphids whose fate is unknown. The general linear model that we used for this statistical analysis was very similar to the analyses presented in the main manuscript, except that we assumed quasibinomial errors.

Results

The analysis of the proportion of parasitoid mummies out of the group of aphids that did not die prematurely showed very similar patterns as that of the number of mummies but with some important quantitative differences. As for the number of mummies, the proportion of mummies differed between aphid backgrounds (Fig S1b, S1 Table; $F_{2,70} = 21.59$, $P < 0.001$) and was affected by the heat treatment (Fig S1a; $F_{2,70} = 9.93$, $P < 0.001$): it was lowest when the aphids experienced heat shock on the day after being parasitized. Again, the presence of X-type had no overall effect on the proportion of mummies ($F_{1,70} = 0.01$, $P = 0.93$), but this differed between the heat treatments ($F_{2,70} = 4.98$, $P = 0.009$). In contrast to the analysis of the number of mummies, the post-hoc tests showed that X-type provided protection from parasitoids for the aphid in the control treatment. However, there was no significant difference between aphids with and without X-type in the two heat treatments. There were also no significant interactions between aphid genotype and the other factors (S1 Table).
